# Enteric nervous system in microbiota-associated gut inflammation

**DOI:** 10.3389/fimmu.2026.1735727

**Published:** 2026-01-27

**Authors:** Cen Yang, Xuping Lan, Huijie Zhong, Jiawei Geng, Wenxue Wang

**Affiliations:** 1 Yunnan University of Chinese Medicine, Kunming, Yunnan, China; 2 Department of Infectious Disease and Hepatic Disease, The First People’s Hospital of Yunnan Province, Kunming, Yunnan, China

**Keywords:** gut inflammation, immune cells, intestinal microbiota, intestinal nerves, microbial dysbiosis

## Abstract

The enteric nervous system (ENS), a distinctive and intricate compartment of the peripheral nervous system (PNS), is characterized by its capacity to autonomously coordinate fundamental gut functions independent of central nervous system (CNS) input. Comprising vast, densely packed networks of neurons and glial cells distributed throughout the intestinal wall, the ENS not only directly governs motility, secretion, and absorption but also engages in dynamic crosstalk with intestinal immune cells to establish immune defense barriers and fine-tune inflammatory responses. This system is persistently exposed to and deeply engaged with a dynamic microenvironment shaped by both external (e.g., microbiota and their metabolites) and internal (e.g., immune cells, stromal cells) signals. The gut microbiota and its metabolic products play pivotal roles in maintaining mucosal barrier integrity and orchestrating the progression of intestinal inflammation. They influence the development and repair of enteric neurons and can directly participate in disease pathogenesis or exert their effects through immune-mediated mechanisms. This review delves into the complex interplay between the ENS and the gut microbiota within the context of intestinal inflammation pathogenesis.

## 1 Introduction

The enteric nervous system (ENS), a crucial component of the autonomic nervous system, represents a highly autonomous neural entity. It constitutes the most extensive component of the peripheral nervous system, with its neural networks fully embedded within the intestinal wall and establishing broad interactions with the endocrine, immune, and central nervous systems, as well as the gut microbiota. As the only hollow organ in the body possessing a complete intrinsic nervous system, the gut can independently execute complex functions without relying on the brain or spinal cord. Owing to its structural complexity, functional autonomy, and significant role in systemic regulation, the ENS is often referred to as the “second brain” (1).

The enteric nervous system (ENS) constitutes a highly organized local neural network equipped with complete functional units. These include intrinsic primary afferent neurons (IPANs) that sense luminal contents, interneurons responsible for information processing, motor neurons that regulate smooth muscle contraction and glandular secretion, as well as enteric glial cells that support and modulate neural function. It is estimated that the adult human gut contains over 500 million neurons (1), which exhibit remarkable diversity in both morphology and neurochemical coding. The ENS is primarily organized into two interconnected plexuses: the myenteric plexus, located between the circular and longitudinal muscle layers, mainly coordinates intestinal propulsive and mixing movements; and the submucosal plexus, situated beneath the mucosal epithelium, primarily regulates ion/fluid secretion, local blood flow, and mucosal barrier function (1), This review will focus particularly on the role of the submucosal plexus in intestinal immune regulation, while also addressing relevant mechanisms of the myenteric plexus in motility control.

Functionally, the ENS innervates nearly all functional units within the gut—including epithelial cells, smooth muscle, interstitial cells of Cajal (ICC), vasculature, and diverse immune cells—enabling it to dynamically sense environmental changes and mount adaptive responses (2, 3). It is particularly noteworthy that the ENS not only operates autonomously but also continuously receives and integrates input from the central nervous system (CNS), forming a multilayered regulatory mechanism (4). Although the fundamental anatomical and physiological framework of the ENS is well-established, the mechanistic details of its interactions with the microbiome and immune system—particularly the molecular and cellular basis of multisystem integration—remain incompletely understood.

Perturbations in ENS signaling compromise epithelial barrier integrity, disrupt immune tolerance, and alter microbiota-host communication, thereby driving colitis pathogenesis through dysregulation of the neuro-immune-microbial axis.

### 1.1 ENS serves as the primary regulator of intestinal motility and secretory function

ENS functions as the central integrative regulator of gastrointestinal motility and secretory activity, coordinating complex sensorimotor programs through a highly specialized neuromodulatory network. Intestinal motility exhibits distinct, spatiotemporally organized patterns: (i) segmental propulsive movements facilitating localized mixing and propulsion; (ii) mixed motor complexes involving blended peristaltic activity; and (iii) migrating motor complexes, coordinated contractile waves propagating aborally across the intestinal tract (1). Regulation of these motility patterns involves a multimodal neuromodulatory network that relies on precisely coordinated excitatory and inhibitory signaling within enteric neuronal circuits. Acetylcholine serves as the principal excitatory neurotransmitter, whereas inhibitory control is mediated by nitric oxide (NO), vasoactive intestinal peptide (VIP), and pituitary adenylate cyclase-activating polypeptide (PACAP) (5). Additional neuromodulators, including 5-hydroxytryptamine (5-HT, serotonin), adenosine triphosphate (ATP), tachykinins such as substance P (SP), and somatostatin, further refine motor outputs through graded modulation of neuronal excitation and smooth muscle contraction (5).

The gastrointestinal tract orchestrates nutrient digestion, absorption, and waste excretion (6) through region-specific specialization along its longitudinal axis. The small intestine mediates primary macronutrient and micronutrient absorption (7), whereas the colon regulates water and electrolyte reabsorption, water-soluble vitamin uptake, and fecal compaction (8). The ENS integrates these region-specific functions through coordinated regulation of motility, secretion, epithelial transport, and immune surveillance. Pathogenic disruptions arise from organisms such *Vibrio cholerae*, rotavirus, and enteropathogenic *E. coli*, whose toxins exploit ENS-driven pathways by activating secretomotor reflexes rather than motor neurons. These responses trigger: (i) uncontrolled fluid secretion via epithelial Cl^-^/HCO_3_^−^ efflux; (ii) disruption of coordinated peristaltic patterns; and (iii) epithelial barrier dysfunction. Collectively, these processes drive ENS-mediated neurogenic inflammation and dysregulated autonomic output, including systemic adrenergic activation via the splanchnic sympathetic pathway, culminating in severe fluid imbalance (9, 10).

Nutritional and mechanical stimuli broadly activate intestinal epithelial cells (11, 12), inducing the release of neuroactive signaling molecules such as 5-HT and cholecystokinin (CCK), which modulate enteric neuronal excitability and dynamically reconfigure intestinal motor patterns (13–15). Microbial metabolites further fine-tune ENS-driven motility via region-specific mechanisms: Bile acids regulate small intestinal transit by prolonging luminal retention to enhance nutrient absorption while simultaneously stimulating high-amplitude propagating contractions in the colon to accelerate microbial metabolite clearance (16, 17). Short-chain fatty acids (SCFAs) maintain physiological motility within optimal concentration thresholds through dual mechanisms: (i) direct activation of enteric neuronal G-protein-coupled receptors (e.g., GPR41/43) and (ii) induction of peptide YY (PYY) from L-cells to suppress colonic motor complexes (18). Notably, motility modulation is strain- and niche-specific across bacterial taxa, with groups such as *Clostridia* and *Proteobacteria* exerting divergent effects on ENS excitability and smooth muscle dynamics, although the underlying neuromodulatory pathways remain incompletely understood.

### 1.2 ENS plays a pivotal role in the pathogenesis and progression of colitis

The ENS, composed of a dense network of enteric neurons and enteric glial cells (EGCs), functions as a dynamic sensor-effector interface capable of detecting and responding to pathophysiological perturbations within the intestinal microenvironment (19). Through this highly adaptive responsiveness, the ENS preserves neural elements and sustains local tissue homeostasis (1, 20, 21), establishing itself as a critical regulator of intestinal reflexes and epithelial barrier function. However, under inflammatory conditions, EGC activity undergoes extensive reprogramming, generating context-dependent effects on neuronal and non-neuronal targets. These responses contribute to a spectrum of outcomes ranging from neuroprotection to neuroinflammation and neurodegeneration (22–24). During acute intestinal inflammation, EGCs often adopt a reactive, pro-inflammatory phenotype that exacerbates neuronal injury, disrupts synaptic remodeling, and impairs neuroplasticity (23, 25, 26). A critical mediator of this process is the overexpression of the EGC-derived Ca^2+^/Zn^2+^-binding protein S100β, which is strongly implicated in the initiation and perpetuation of intestinal inflammation in the human colon (27). At nanomolar concentrations, S100β promotes neuronal survival and neurite outgrowth (24). In contrast, at higher levels, it enhances mucosal macrophage recruitment and drives inflammatory amplification through activation of pro-inflammatory signaling cascades tightly linked to the severity of neurodegenerative and epithelial injury (24, 28).

However, recent studies have challenged the presumed specificity of S100β expression in glial cells, revealing its low-level expression in certain immune cells as well (29). Whether this cellular heterogeneity in S100β expression differentially influences intestinal inflammation remains unclear. Furthermore, the same study proposed a novel glia–Paneth cell interaction model, demonstrating that mucosal glial cell depletion impairs Paneth cell secretory function and subsequently alters gut microbial composition (29). This mechanism may indirectly enhance intestinal susceptibility to inflammation, providing compelling evidence for the bidirectional regulation of intestinal inflammation through ENS–microbiota crosstalk.

**Figure 1:**
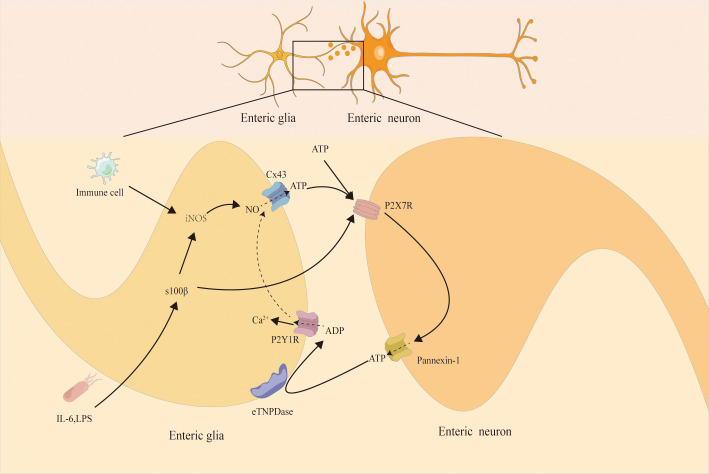
The activation of neuronal P2X7 receptors (P2X7Rs) induces ATP release through the pannexin-1 (panx1) channel. This process triggers the response of peripherally located glial cells, which activate glial cell P2Y1 receptors (P2Y1Rs). Activation of P2Y1R triggers downstream intracellular signaling pathways to drive glial cells and thereby induce the production of nitric oxide synthase (iNOS). In addition, intestinal neuroglial cells mediate NO-dependent inflammation through S100B expression and release, thereby triggering the production of inducible nitric oxide synthase (iNOS) and nitric oxide. Meanwhile, S100B can induce ATP release through the semi-channel of connexin 43 (Cx43) by opening. The nitric oxide (NO) released by glial cells can accelerate the ATP release through the Cx43 semi-channel-dependent manner. Possibly, the NO produced by glial cells can also act back on neurons and inhibit the activity of the panx1 channel. A large amount of ATP is released through the Cx43 channel of glial cells and acts on neuronal P2X7Rs, ultimately leading to neuronal death.

EGCs express a diverse repertoire of purinergic and adenosine receptors, including P2Y1 (30–32), P2Y4 (33–35), and A2B receptors (36, 37), alongside the ectoenzyme ectonucleoside triphosphate diphosphohydrolase 2 (eNTPDase2). These receptors actively participate in inflammatory responses and interact with pro-inflammatory mediators. In chronic intestinal inflammatory diseases such as Crohn’s disease, celiac disease, and ulcerative colitis (UC), EGCs drive S100β overexpression and potentiate nitric oxide (NO)-dependent inflammation (28, 38). This process involves activation of the inducible nitric oxide synthase (iNOS) pathway, resulting in elevated NO production, increased oxidative stress, and progressive neuronal injury (39). Furthermore, opening of connexin 43 (Cx43) hemichannels facilitates ATP efflux from reactive EGCs (22), driving feedforward signaling loops in which extracellular ATP stimulates neuronal P2X7 receptors, pannexin-1 (Panx1) channels, and the connexin/ASC (apoptosis-associated speck-like protein containing a CARD) inflammasome complex. These interactions converge on pathways that mediate neuronal cell death and amplify local neuroinflammation (31) (**Figure 1**). Although the influence of EGCs remains context dependent—exerting neuroprotective effects under homeostatic conditions while promoting tissue injury under sustained inflammatory stress—the mechanisms governing these transitions remain incompletely resolved. Elucidating the molecular programs underlying EGC plasticity and signaling holds significant therapeutic potential for modulating ENS-driven neuroimmune crosstalk and developing targeted interventions for chronic intestinal inflammatory disorders such as ulcerative colitis.

### 1.3 Neuroimmune axis: crucial mediator of ENS regulation in colitis

The ENS critically regulates intestinal inflammation through bidirectional interactions with immune circuits, collectively forming the neuroimmune axis (40, 41). This axis integrates diverse cellular and molecular components, including enteric neurons, vagal afferents, and vagal efferents, coordinating immune responses and epithelial homeostasis. Notably, vagal efferent pathways play a pivotal anti-inflammatory role by establishing a cholinergic reflex loop between enteric neurons and muscularis macrophages (42). Activation of vagal efferents stimulates cholinergic neurons within the myenteric plexus, triggering acetylcholine (ACh) release onto muscularis macrophages, which inhibits ATP-induced Ca^2+^ flux, suppresses macrophage activation, and limits pro-inflammatory cytokine release (41). The study by Matthews et al. further elucidates a specific neuroprotective mechanism during infection (43). They demonstrated that *Salmonella*-induced colitis preferentially damages glutamatergic myenteric neurons expressing certain atypical inflammasomes. Crucially, tissue-resident macrophages surrounding enteric neurons were found to effectively counteract inflammation-driven neurodegeneration via the arginase-1–polyamine axis. This indicates that macrophages can actively modulate local inflammatory responses to preserve enteric nervous system function (44, 45). Their work not only reinforces the classical understanding that macrophages exert neuroprotective effects but, more importantly, identifies a concrete neuro-immune regulatory pathway in the context of infectious colitis. These findings provide key mechanistic insights into how the nervous system actively participates in inflammatory recovery.

ENS-immune communication is further shaped by neuropeptide-mediated signaling. VIP, released from enteric neurons, contributes to the regulation of inflammatory processes (46). Following microbial infection, group 3 innate lymphoid cells (ILC3s) upregulate expression of VIP receptor 2 (VIPR2), enabling targeted responsiveness to VIP signaling (47). VIP stimulation of ILC3s promotes secretion of interleukin-22 (IL-22), a tissue-protective cytokine that enhances epithelial regeneration and barrier repair (48). In contrast, SP, a highly conserved neuropeptide enriched within enteric circuits, facilitates immune cell proliferation and cytokine release. Clinical studies have reported increased densities of SP-positive neurons in the myenteric plexus of patients with UC and inflammatory bowel disease (49–51), suggesting SP−driven amplification of inflammatory pathways. Mast cells exhibit close anatomical and functional associations with intestinal neurons (52) and respond to neuron-derived SP and VIP through degranulation and cytokine production (53). Reciprocally, mast cell-derived mediators activate intestinal neurons, inducing neuronal hyperexcitability (54–56), potentially precipitating neurodegeneration, neuroinflammation, and ultimately neuronal cell death. Although neuronal density frequently correlates with inflammatory burdens, the relationship between neurodegeneration and intestinal inflammation remains complex. However, the oversimplified linear paradigm that equates “neurodegeneration with harm” fails to explain the complex phenomena observed in this field. Recent research has revealed that Sarm1-mediated degeneration of catecholaminergic axons in the colon does not exacerbate disease but rather alleviates colitis by disrupting local pro-inflammatory signaling pathways (57). This finding suggests that, under specific conditions, neurodegeneration may represent an active, protective adaptation initiated by the host to limit inflammatory damage. These insights further demonstrate that the “enteric nervous system” cannot be regarded as a homogeneous entity; instead, distinct neuronal subtypes may play markedly different—even opposing—roles within the inflammatory microenvironment.

## 2 Gut microbiota dysbiosis as a key contributor to colitis pathogenesis

### 2.1 Intestinal microecological homeostasis: a fundamental determinant of mucosal immunity

The gastrointestinal tract hosts a highly diverse and dynamic community of commensal microorganisms, including bacteria, viruses, fungi, and protozoa (58). This community undergoes continuous fluctuations driven by host diet, pharmacological interventions, and pathological states, with the colon harboring the greatest microbial density and taxonomic complexity. Dominant bacterial phyla, including *Bacteroidetes*, *Firmicutes*, *Actinobacteria*, and *Proteobacteria*, exhibit relative compositional stability under physiological conditions but display rapid adaptive responses to environmental perturbations. The gut microbiota critically regulates epithelial barrier integrity through modulation of tight junction architecture. For instance, *Lactobacillus plantarum* preserves the structural stability of tight junction proteins, thereby preventing barrier collapse and attenuating epithelial dysfunction (59). Loss of barrier integrity facilitates translocation of luminal bacteria, endotoxins, dietary antigens, and metabolic waste into the systemic circulation, activating maladaptive immune responses that drive mucosal inflammation. Clinically, these disruptions manifest as visceral hypersensitivity, dysmotility, bloating, and food intolerances, aligning with the pathological framework of intestinal hyperpermeability syndrome (“leaky gut”), which may progress to hemorrhagic diarrhea in severe colitis (60).

The gut microbiota plays an indispensable role in shaping effector T cell responses within the gastrointestinal mucosa. Within this context, Th17 cells constitute a distinct subset of helper CD4^+^ T lymphocytes characterized by their capacity to secrete IL-17A, IL-17F, IL-21, and IL-22 (61). IL-17A functions as a potent pro-inflammatory cytokine, synergizing with tumor necrosis factor-α (TNF-α) and IL-1β to amplify inflammatory cascades across epithelial, stromal, and immune compartments (62). Dysregulation of Th17 activity has been implicated in the pathogenesis of inflammatory and autoimmune disorders, including colitis. Germ-free mouse models demonstrate marked reductions in intestinal Th1 and Th17 populations, with restoration occurring only following recolonization by conventional microbiota (63), underscoring the indispensable role of commensal communities in T cell lineage development. Consequently, maintaining microbial diversity and ecological balance constitutes a fundamental requirement for sustaining intestinal immune competence and preventing chronic mucosal inflammation.

In addition to shaping adaptive immunity, the gut microbiota regulates the induction and maturation of gastrointestinal immune components, thereby regulating immune homeostasis and inflammatory responses (64, 65). Conversely, the mucosal immune system exerts reciprocal control over microbial composition and metabolic function (64). While this tightly regulated mutualism maintains intestinal health, its disruption precipitates profound immunological and metabolic disturbances, contributing to both localized gastrointestinal disease and systemic pathology. Notably, the gut microbiota also modulates gut-brain-axis communication via multiple mechanisms. Microbial-derived metabolites—including SCFAs, γ-aminobutyric acid (GABA), tryptophan derivatives, 5-HT, and catecholamines—serve as key signaling molecules that regulate intestinal resident cells (66, 67). These metabolites additionally act through endocrine pathways to influence distant organ systems, linking intestinal microbial dynamics to systemic neuroimmune regulation. Consequently, dysbiosis can fundamentally perturb gut-brain signaling, impairing local mucosal immunity while driving widespread immune dysregulation that contributes to colitis pathogenesis.

### 2.2 Intestinal dysbiosis disrupts immune homeostasis and directly contributes to colitis

The gut microbiota is essential for sustaining intestinal health by regulating epithelial barrier integrity (68) and modulating local and systemic immune function, often promoting a pro-inflammatory environment (68). Perturbations in microbial composition can arise from dietary imbalances, excessive pharmacotherapy, alcohol consumption, smoking, and chronic stress, leading to the depletion of beneficial bacteria and overgrowth of pathogenic species (69), thereby disrupting immune homeostasis. Dietary patterns represent particularly potent modulators of inflammation via their impact on microbial composition. High-fat/high-sugar diets induce compositional shifts characterized by expansion of pro-inflammatory taxa and depletion of protective species, resulting in impaired gastrointestinal mucosal function (70). In contrast, omega-3 polyunsaturated fatty acids promote enrichment of beneficial taxa, enhance the production of SCFAs, and reduce gastrointestinal permeability, although evidence regarding their immunomodulatory effects remains inconclusive (71). Conversely, high-carbohydrate, low-fiber diets consistently associate with adverse alterations in gut transit, bacterial diversity, and SCFA biosynthesis (72–74), collectively compromising mucosal homeostasis. Furthermore, micronutrient deficiencies can also reduce overall bacterial abundance and favor the proliferation of potentially pathogenic species such as *Escherichia coli* (75). Notably, excessive micronutrient intake may exacerbate infection susceptibility; for instance, iron supplementation in infants has been shown to promote colonization by enteropathogenic *E. coli* and aggravate intestinal inflammation (76, 77). These findings underscore the necessity of maintaining balanced dietary habits and adequate nutrient intake to preserve commensal stability and maintain epithelial and immune homeostasis.

Beyond dietary factors, pharmacological interventions constitute another critical driver of microbial dysregulation, with antibiotic misuse representing the most significant contributor (78, 79). Experimental studies in murine models have demonstrated that early-life antibiotic exposure disrupts microbiome assembly, markedly depleting species- and strain-level diversity. These perturbations precipitate metabolic programming, contributing to adiposity, skeletal dysregulation, and impaired immune maturation (80). Specific antibiotics induce distinct taxonomic disruptions: amoxicillin promotes expansion of *Enterobacteriaceae* (81) and enrichment of pathogenic genera such as *Enterococcus*, *Staphylococcus*, and *Streptococcus* (82), while simultaneously depleting health-promoting taxa including *Blautia*, *Collinsella*, *Oscillospira*, and *Roseburia* (83). Quinolone administration exerts heterogeneous effects on gut ecosystems by increasing the relative abundance of *Bacteroides*, *Proteobacteria*, and select *Clostridiales*, coupled with reduced representation of *Faecalibacterium* (84–86). Tetracycline treatment elevates *Bacteroidetes* prevalence while decreasing *Bifidobacterium*, *Lactobacillus*, *Bacteroides fragilis*, and *Enterococcus* populations (87). Collectively, antibiotic exposure induces profound and often long-lasting restructuring of gut microbial consortia, compromising barrier function and immune homeostasis (88). Nevertheless, numerous non-antibiotic therapeutics exert downstream effects on microbiota composition and metabolic activity. Metformin induces enrichment of *Akkermansia muciniphila* and other SCFA-producing taxa (89), potentially influencing host metabolic pathways and mucosal function. In contrast, mycophenolate mofetil (MMF) drives pronounced α-diversity loss, characterized by increased representation of pathogenic *Escherichia/Shigella* and depletion of protective genera such as *Clostridium*, *Akkermansia*, and *Parabacteroides*, correlating with rapid weight loss and exacerbated colonic inflammation in experimental models (90). Moreover, alcohol consumption and tobacco smoking drive extensive, well-documented disruptions of gut microbial composition, enhancing intestinal permeability and amplifying inflammatory responses.

### 2.3 Microbial metabolites mediate gut microbiota regulation of colitis

Gut microbiota-derived metabolites serve as critical signaling molecules linking dietary inputs, microbial activity, and host immune responses in colitis pathogenesis. Dietary substrates undergo microbial fermentation to produce diverse bioactive compounds, including SCFAs, branched-chain fatty acids, ammonia, hydrogen sulfide (H_2_S), indoles, phenolic derivatives, and other signaling metabolites (91, 92). Short-chain fatty acids (SCFAs) generate neural signals by stimulating the production of 5-HT in enterochromaffin cells via the GPR41 receptor, thereby regulating intestinal muscular motility while concurrently exerting neuroprotective effects (93). Among these, SCFAs play a central role in maintaining intestinal immune homeostasis by promoting regulatory T-cell differentiation through upregulation of the FoxP3 transcription factor (92, 94). In contrast, proteolytic fermentation generates potentially harmful metabolites such as ammonia and p-cresol, which compromise epithelial integrity and disrupt barrier function (92, 94). Bile acid-derived metabolites represent another critical regulatory axis, modulating inflammatory cytokine expression through activation of the intestinal farnesoid X receptor (FXR) and Takeda G protein-coupled receptor 5 (TGR5) signaling pathways (95, 96). Taurine-conjugated H_2_S demonstrates paradoxical, context-dependent effects on intestinal homeostasis, inhibiting colonocyte β-oxidation of SCFAs, thus impairing energy metabolism and barrier function (97). Notably, *Lactobacillus reuteri* converts tryptophan into indole-3-aldehyde, which activates aryl hydrocarbon receptor (AhR)-dependent IL-22 expression, significantly ameliorating dextran sulfate sodium (DSS)-induced colitis symptoms in murine models. Meanwhile, the aryl hydrocarbon receptor (AhR) is specifically highly expressed in myenteric neurons of the colon, where it modulates intestinal motility through transcriptional regulation of CYP1A1 (98).

Members of the *Lactobacillus* genus exert additional metabolite-mediated protective effects. Notably, *Lactobacillus* species constitutively produce nanomolar concentrations of hydrogen peroxide (H_2_O_2_), providing broad-spectrum defense against invasive pathogens (99). In murine DSS-colitis models, *Lactobacillus*-derived H_2_O_2_ enhances mucosal cytoprotection and accelerates epithelial repair (100). Additionally, *Lactobacillus* species metabolize dietary lipids into bioactive oxylipins and hydroxy fatty acids that influence host metabolic and inflammatory pathways (101, 102). While microbial metabolites represent essential mediators of microbiota-host crosstalk, their functional impact depends substantially on dietary substrate availability from host nutrition. Given the intermittent nature of nutrient delivery during postprandial and fasting periods, metabolite production exhibits circadian oscillations that limit sustained homeostatic regulation. Consequently, constitutively produced microbial signaling molecules may exert greater pathophysiological relevance. These include: (i) microbial-associated molecular patterns (MAMPs, e.g., lipopolysaccharides (LPS), peptidoglycan); (ii) bacteriocins and quorum-sensing molecules; and (iii) constitutively secreted metabolites such as H_2_O_2_. Disruption of microbial community stability, particularly alterations in bacterial populations specializing in tonic production of these bioactive compounds, likely represents a key determinant of intestinal homeostasis dysregulation and heightened susceptibility to colitis.

## 3 Gut microbiota-ENS crosstalk in colitis pathogenesis

### 3.1 Bidirectional signaling between microbial communities and ENS function

The ENS operates as a dynamic integrative hub enabling continuous bidirectional communication between the gut microbiota and the CNS, thereby coordinating neural, immune, and metabolic responses. Owing to their anatomical proximity, gut microbial communities exert profound direct and indirect effects on ENS development, structural organization, and functional plasticity. These interactions are mediated through diverse signaling cascades involving microbial-derived components and metabolites and host-derived immune and neuroactive mediators, which collectively regulate enteric neuronal excitability and gastrointestinal motility. Postnatal colonization of the gastrointestinal tract by a complex microbial ecosystem profoundly shapes host physiology, impacting energy homeostasis, immune responses, behavioral regulation, and circadian rhythm entrainment (103–105). Evidence from germ-free mouse models demonstrates an essential role for the microbiota in ENS maturation and function, with the absence of commensal colonization leading to reduced enteric neuronal density, impaired intestinal peristaltic reflexes (106–108), and defective recruitment of EGCs into the intestinal mucosa during development, ultimately compromising neuroimmune integration and homeostatic regulation (109). These findings underscore the critical contribution of microbiota-driven signals to proper assembly and functional signaling of intestinal neural circuits. Notably, recent studies have indicated that interactions between microbial communities and ENS development may commence earlier than previously thought, potentially during gestation via maternal microbial metabolites and immune factors transferred through the uteroplacental circulation (110, 111). Maternal microbial-derived signaling molecules and immune mediators act in concert to shape fetal ENS development, influencing offspring neuroimmune competence and intestinal homeostasis. Consequently, disruption of maternal or early-life microbial inputs may predispose individuals to long-term intestinal immune dysregulation and ENS dysfunction, increasing susceptibility to neurodevelopmental disorders and inflammatory pathologies later in life.

**Figure 2:**
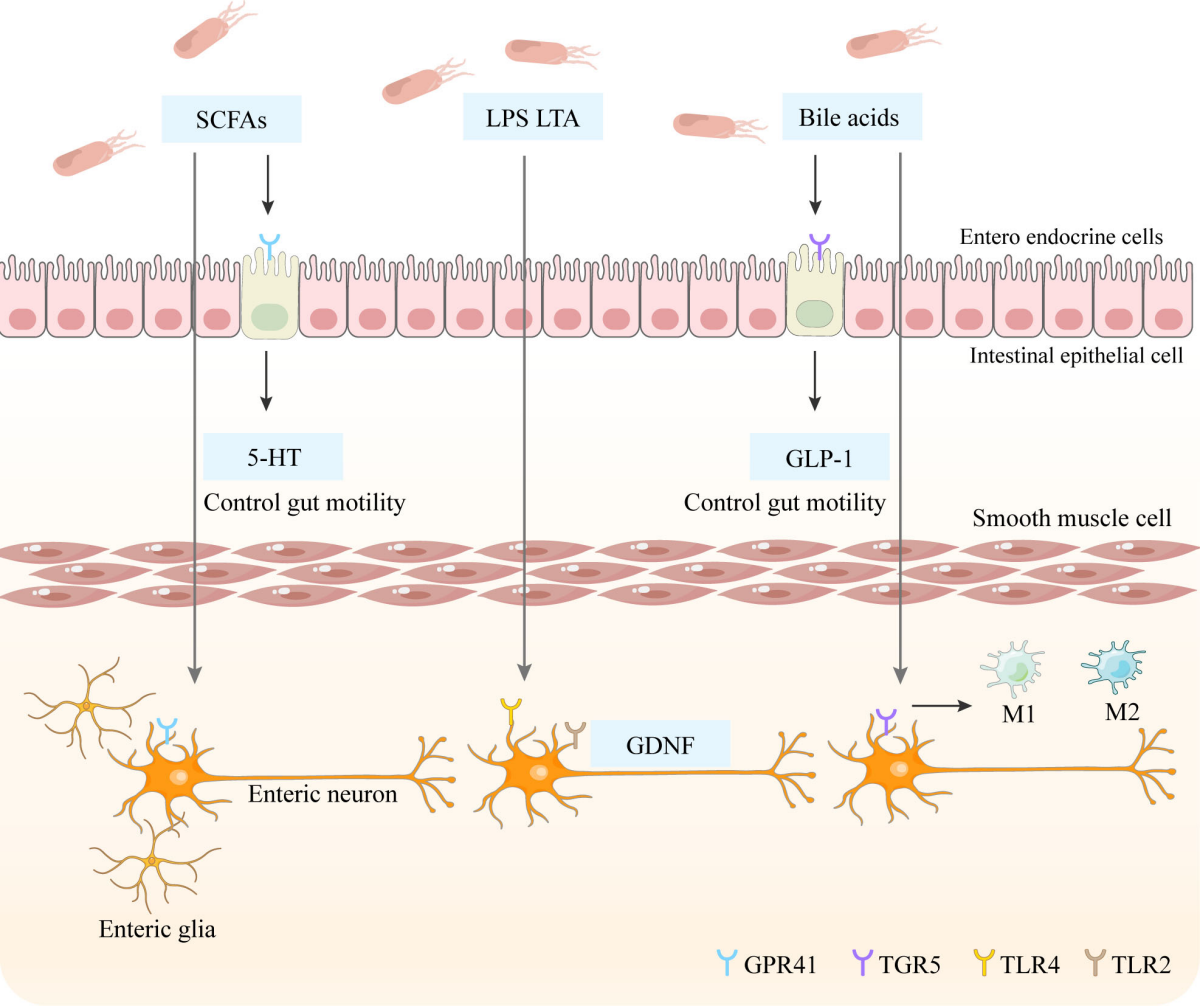
The SCFA will activate the GPR41 receptors on the entero-endocrine cells, thereby promoting the generation of 5-HT to control gut motility. Additionally, the SCFA will also act on the Enteric glia and GPR41 receptors on the Enteric neuron to change the gut motility. 2. LTA and LPS can directly act on the TLR2 receptors on smooth muscle cells and can maintain the subgroups of Enteric neurons and affect intestinal peristalsis. At the same time, they can also act on the TLR4 and TLR2 receptors on Enteric neurons, generating neurotrophic factors (GDNF). GDNF can maintain the number of neurons and promote the normal development of neurons. 3. Bile acids directly act on the TGR5 receptor, thereby stimulating the release of GLP1. GLP1 can stimulate insulin secretion to inhibit appetite and slow down intestinal peristalsis. At the same time, it can also act on the TGR5 receptors on the Enteric neuron to let M1 become M2.

Critically, restoration of gut microbiota in germ-free models reverses both structural and functional abnormalities of the ENS (112–114). This recovery is primarily mediated by Toll-like receptor (TLR) signaling pathways, which sense specific bacterial cell wall components and integrate microbial cues into neuronal development. TLR2 recognizes lipoteichoic acid, whereas TLR4 responds to LPS (115). Mechanistic studies have revealed that TLR2 deficiency selectively reduces ileal neuronal density, an effect fully reversed by TLR2 agonist stimulation. Studies using longitudinal muscle-myenteric plexus (LMMP) preparations isolated from TLR2−/− mice in an organ culture system demonstrated that deficiency in TLR2—which primarily recognizes lipoteichoic acid (LTA)—led to reduced enteric neuronal density, accompanied by neuromuscular dysfunction, and increased morbidity and mortality during inflammatory challenge. Furthermore, administration of a TLR2 agonist was shown to restore enteric neuron numbers and attenuate the severity of colitis (116). Similarly, TLR4 knockout induces ENS hypoplasia, characterized by marked depletion of nitrergic neurons within the colonic myenteric plexuses and demonstrates decreased survival of cultured neurons following LPS exposure (108) (**Figure 2**). Furthermore, antibiotic-induced dysbiosis in adult mice with an established ENS triggers apoptosis of enteric neurons (108), although the precise cellular targets mediating this process remain unidentified.

Beyond TLR-dependent mechanisms, gut microbial communities exert significant regulatory control over mucosal 5-HT production (117, 118). While 5−HT is widely recognized for its pro-inflammatory properties (119, 120), activation of 5-HT receptors in the ENS demonstrates context-dependent neuroprotective effects, promoting adult neurogenesis, preserving neuronal viability (93), and supporting the maturation and survival of dopaminergic neurons (**Figure 2**). Collectively, these findings highlight how microbial communities orchestrate ENS ontogeny and adaptive remodeling, from progenitor differentiation to functional circuit integration, establishing a finely tuned bidirectional communication network critical for maintaining gastrointestinal homeostasis and barrier integrity.

### 3.2 Microbial metabolite regulation of ENS activity

Microbiota-derived metabolites, particularly SCFAs and bile acids, play essential roles in modulating ENS activity through direct and indirect neuromodulatory mechanisms. Primary bile acids undergo microbial biotransformation, primarily through bile salt hydrolase-mediated deconjugation (121). Dysbiosis disrupts these processes, impairing both bile acid hydrolysis and 7α-dehydroxylation pathways, which alters the physiological balance between primary and secondary bile acids (122). These metabolites exert significant effects on intestinal motility patterns: secondary bile acids accelerate colonic transit while paradoxically slowing small intestinal propulsion (16, 17), a differential outcome likely mediated by glucagon-like peptide-1 (GLP-1) secretion, which inhibits gastrointestinal motility (123). TGR5, which is highly expressed on enteric neurons (124), demonstrates ligand-specific activation (LCA > DCA > CDCA > CA) (125). Notably, TGR5 agonism reduces pro-inflammatory cytokines, including IL-6, IFN-γ, and TNF-α, while enhancing anti-inflammatory IL-10 production (126, 127), and promotes macrophage polarization toward an anti-inflammatory phenotype (126, 127) (**Figure 2**). However, elevated concentrations of primary bile acids exacerbate epithelial cytotoxicity and colitis severity (128), revealing a concentration-dependent paradox in bile acid signaling. The complex interactions among bile acid composition, mucosal inflammation, and microbial ecology remain incompletely characterized, limiting definitive conclusions regarding TGR5-mediated effects across physiological and pathological contexts.

Microbial fermentation of dietary substrates yields SCFAs, among which acetate, propionate, and butyrate are particularly prominent and functionally relevant (129). These metabolites serve as central mediators of microbiota-ENS communication. Within the ENS, SCFAs are actively sensed by enteric neurons; for example, butyrate enters neuronal cytoplasm via monocarboxylate transporter 2 (MCT2) (130). Once internalized, SCFAs significantly increase the proportion of intestinal neurons expressing choline acetyltransferase (ChAT), thereby enhancing cholinergic neurotransmission and promoting colonic circular muscle contraction and intestinal peristalsis (131). However, *ex vivo* analysis of isolated colonic tissue has revealed distinct substrate-specific effects on motility: butyrate accelerates fecal propulsion, while propionate suppresses it (132), indicating functional divergence among SCFA species. Beyond direct modulation of neuronal activity, SCFAs influence neuroepithelial signaling through actions on enteroendocrine cells, which express multiple G protein-coupled receptors (GPCRs), including free fatty acid receptors 2 (FFAR2) and FFAR3 (also known as GPR41), enabling SCFA-driven hormone and neuropeptide release. Importantly, FFAR3 is also expressed by a subset of enteric neurons themselves (133), suggesting dual direct and indirect pathways of SCFA-mediated ENS regulation. Whether SCFAs predominantly modulate ENS activity via indirect mechanisms—through EEC-derived hormones and neuropeptides—or through direct receptor-mediated signaling across the epithelium remains unresolved. It is plausible that both routes operate in parallel, with spatial, temporal, and metabolite-specific dynamics shaping functional outcomes.

### 3.3 Intestinal microbial regulation of the neuroimmune axis represents a pivotal driver of colitis pathogenesis

In the complex intestinal microenvironment, innate lymphoid cells (ILCs) and neurons form an intricate neuro-immune regulatory network that dynamically coordinates immune defense and tissue homeostasis. This network plays a particularly critical role in regulating ILC3s. Upon food intake, activated VIP-positive neurons release vasoactive intestinal peptide (VIP), which acts directly on ILC3s to suppress IL-22 production, thereby transiently dampening immune responses to prioritize nutrient absorption (48). Conversely, enteric glial cells enhance ILC3-derived IL-22 secretion and reinforce barrier function via the neurotrophic factor–RET signaling axis (134). Furthermore, GABAergic neurons release γ-aminobutyric acid (GABA) to inhibit ILC3 proliferation and the production of the pro-inflammatory cytokine IL-17A, constituting a crucial “braking” pathway that prevents intestinal inflammation (135).

Likewise, ILC2s are subject to stringent neuronal control. The neuropeptide α-CGRP suppresses excessive ILC2 expansion (136), while adrenergic signaling negatively regulates ILC2 responses and limits type 2 inflammation through the β2-adrenergic receptor (β2AR) (137). Together, these findings illustrate how the nervous system exerts precise, switch-like control over distinct ILC subsets via diverse neurotransmitters and neuropeptides in a context-dependent manner. Dysregulation of this sophisticated cross-talk represents a key mechanism in the pathogenesis of inflammatory bowel diseases and offers promising targets for novel therapeutic interventions.

Macrophages embedded within the intestinal muscularis externa—characterized by the distinct (CD103^+^ and CD11b^+^)phenotype and dependent on colony-stimulating factor 1 (CSF1) signaling (45, 138)—maintain reciprocal interactions with the ENS. Intestinal neurons expressing bone morphogenetic protein 2 (BMP2) receptors secrete CSF1 to support the development and maintenance of muscularis macrophages. In CSF1-deficient mice, ENS architecture becomes disorganized and hyperganglionosis emerges, underscoring the role of neuron-derived CSF1 in sustaining neuromuscular homeostasis (45).

Complementary to this, EGCs also serve as primary mediators of neuroimmune dynamics through CSF1 secretion (19, 31, 39, 139). Both clinical and experimental data have increasingly implicated neuroinflammation as a key contributor to visceral hypersensitivity (138), with bidirectional nociceptor-EGC communication initiating and amplifying pro-inflammatory cascades (23). Inflammatory stimuli disrupt EGC function, as evidenced by altered expression of connexins. Specifically, inflammation suppresses Cx43 while inducing alternative isoforms, thereby impairing Cx43 hemichannel-dependent release of macrophage colony-stimulating factor—a putative mechanism underlying post-inflammatory intestinal hypersensitivity (139). Paradoxically, although glial-derived S100β promotes neuronal remodeling through Cx43-dependent mechanisms under homeostatic condition, its overexpression during intestinal inflammation triggers widespread glial activation, thereby exacerbating neuroinflammation and neuronal damage (31). Shifts in microbial ecology may further exacerbate this process by modulating S100β expression or disrupting glial-neuronal communication networks via bacterial toxins or metabolites. While the precise molecular drivers remain incompletely defined, accumulating evidence points to microbiota-glia-neuron interactions as a critical nexus sustaining chronic visceral hypersensitivity and intestinal inflammation.

## 4 Discussion

The enteric nervous system (ENS), often referred to as the “second brain,” not only autonomously regulates local intestinal physiological functions but also engages in bidirectional communication with the central nervous system (CNS) via the gut–brain axis. The primary pathways through which the ENS transmits signals to the CNS include: vagal and spinal afferent pathways that relay sensory information from the gut to the brain; hormones released by enteroendocrine cells (e.g., 5-HT and GLP-1) that act on brain regions via the bloodstream; and immune mediators that influence CNS function by crossing the blood–brain barrier or acting through neural pathways. This bottom-up communication enables the ENS to contribute not only to the maintenance of intestinal homeostasis but also to the pathophysiology of CNS disorders such as anxiety, depression, and neurodegenerative diseases.

Building on this foundation, we further focus on the specific mechanisms underlying the microbiota–immune–ENS triad in the context of intestinal inflammation such as colitis. Current therapeutic strategies for colitis predominantly target immune modulation, often overlooking the pivotal role of the ENS in both disease initiation and repair. Although enteric neuronal damage was long considered irreversible, emerging evidence indicates that the adult ENS retains a considerable capacity for regeneration and remodeling. Neurotrophic factors (e.g., GDNF), 5-HT, and glial-derived signals have been shown to promote neurogenesis and circuit reorganization, offering novel avenues for ENS-targeted interventions in colitis.

Modern lifestyle factors, including chronic stress and dietary irregularities, contribute to intestinal dysbiosis, which not only disrupts the epithelial barrier and immune balance but also directly influences ENS neurons and glia via microbial metabolites (e.g., short-chain fatty acids and GABA). Such interactions can alter neuronal excitability and neurotransmitter release, and may even lead to irreversible neuronal injury. ENS dysfunction, in turn, exacerbates disturbances in intestinal motility, secretion, and immune regulation, creating a vicious cycle that promotes chronicity and treatment resistance.

Future studies should aim to elucidate the dynamic regulatory network formed by the microbiota–immune–ENS triad during the initiation and progression of colitis. Key priorities include clarifying the precise mechanisms by which specific microbial metabolites modulate ENS cellular phenotypes and plasticity; defining the temporal features of signaling crosstalk between the ENS and local immune cells during inflammation; and identifying critical endogenous and exogenous signals that promote ENS repair within an inflammatory milieu. Unraveling these aspects is expected to facilitate the development of multi-target therapeutic strategies directed at the neuro–immune–microbial network, opening new pathways for the prevention and treatment of colitis and other gut–brain axis-related disorders.
